# The Challenges Toward Real-world Implementation of Digital Health Design Approaches: Narrative Review

**DOI:** 10.2196/35693

**Published:** 2022-09-09

**Authors:** Anthony Duffy, Gregory J Christie, Sylvain Moreno

**Affiliations:** 1 School of Interactive Arts & Technology Simon Fraser University Surrey, BC Canada; 2 Digital Health Circle Surrey, BC Canada

**Keywords:** digital health, end users, user experience, health behavior, intervention, co-design, mobile health, mobile phone

## Abstract

**Background:**

Digital health represents an important strategy in the future of health care delivery. Over the past decade, mobile health has accelerated the agency of health care users. Despite prevailing excitement about the potential of digital health, questions remain on efficacy, uptake, usability, and patient outcome. This challenge is confounded by 2 industries, digital and health, which have vastly different approaches to research, design, testing, and implementation. In this regard, there is a need to examine prevailing design approaches, weigh their benefits and challenges toward implementation, and recommend a path forward that synthesizes the needs of this complex stakeholder group.

**Objective:**

In this review, we aimed to study prominent digital health intervention design approaches that mediate the digital health space. In doing so, we sought to examine the origins, perceived benefits, contrasting nuances, challenges, and typical use-case scenarios of each methodology.

**Methods:**

A narrative review of digital health design approaches was performed between September 2020 and April 2021 by referencing keywords such as “digital health design,” “mHealth design,” “e-Health design,” “agile health,” and “agile healthcare.” The studies selected after screening were those that discussed the design and implementation of digital health design approaches. A total of 120 studies were selected for full-text review, of which 62 (51.6%) were selected for inclusion in this review.

**Results:**

A review identifying the 5 overarching digital health design approaches was compiled: user-centered design, person-based design, human-centered design, patient-centered design, and patient-led design. The findings were synthesized in a narrative structure discussing the origins, advantages, disadvantages, challenges, and potential use-case scenarios.

**Conclusions:**

Digital health is experiencing the growing pains of rapid expansion. Currently, numerous design approaches are being implemented to harmonize the needs of a complex stakeholder group. Whether the end user is positioned as a person, patient, or user, the challenge to synthesize the constraints and affordances of both digital design and health care, built equally around user satisfaction and clinical efficacy, remains paramount. Further research that works toward a transdisciplinarity in digital health may help break down friction in this field. Until digital health is viewed as a hybridized industry with unique requirements rather than one with competing interests, the nuances that each design approach posits will be difficult to realize in a real-world context. We encourage the collaboration of digital and health experts within hybrid design teams, through all stages of intervention design, to create a better digital health culture and design ethos.

## Introduction

### Background

With an estimated 1.7 billion smartphone users downloading health care apps in 2018 [1], digital health represents an important strategy in the future of health care delivery [2]. The field represents an emerging sociotechnical [3] design space that fuses together health care, the digital industry, and academia. The rapid growth of digital technologies has shifted digital health from internet-based apps for medical content, commerce, and connectivity to a broad spectrum of emerging, always-on technologies such as genomics, artificial intelligence, wearables, mobile apps, and telemedicine [4]. Self-management is becoming a cornerstone of the health system [5]. With this, the complexity of digital health interventions (DHIs) has increased [6], presenting vast variance in use-case scenarios that reach beyond typical health validation or technical usability approval. Digital health poses the unique design challenge of digital and health professionals collaborating in a multistakeholder environment with very disparate methodologies on how to design a solution. This new ecosystem brings new and complex challenges to the design ethos.

The importance of new approaches to digital health is highlighted by the Food and Drug Administration requirement for end-user involvement in validating the design process for usability and human factors [7]. In addition, in 2018, the World Health Organization developed a detailed taxonomy of digital health [4], accentuating its rapid expansion. Despite receiving US $6 billion in funding in 2017 [4], concerns regarding uptake [8], usability [9], and patient outcomes [6] continue to confound digital health.

### Context

To address these concerns, numerous design approaches are being proposed today. However, a key challenge to overcome is the variance in perspective among health experts, user experience (UX) designers, patients, academics, etc. Where designers may lack a theoretical basis and clinical foundation, health experts may lack knowledge of agile development methodologies and UX design [10] and academics often navigate both spaces, seeking to develop common ground. Dovetailing various specialists from 2 distinctly different mindsets is at the root of the challenge [11]. Simply layering on industry agile design approaches to traditional health care intervention design has proven problematic. The definition of measurable outcomes [12] is a lengthy process in health care. By contrast, validating outcomes in the digital industry is an iterative process that is not bound to a long-term expansive data set. From a digital perspective, usability is premised on user validation and satisfaction; from a health care perspective, usability is premised on safety and clinical efficacy. The merging of digital and health into one ecosystem challenges the incentivization of both partners [12]. Therefore, in spite of technologies that have given rise to exciting new forms of health interventions (ie, sensory apps and wearables), patient outcomes are difficult to measure because of the disparity in the evaluation methods of slow, safe, and scientific evaluation in health care and rapid, lean, and iterative evaluation in the digital industry [1]. For example, a health app may be validated on UX design principles evaluating qualitative feedback regarding user efficacy. However, it may be invalidated by health safety and clinical efficacy trials, showing no therapeutic benefit. Similarly, a health app may pass rigorous, quantitative health-based trials but receive no uptake because of a failure to validate the UX based on sound design principles. Furthermore, there is the additional layer of variance in health regulation at federal and local levels. Understanding that designing positive patient outcomes in digital health is a blend of both health improvement and successful user engagement is part of the path forward.

### Objectives

In seeking to resolve this problem, a better understanding of digital health design approaches is needed for improving use-case effectiveness, for potential hybridization of methods, and overall to reduce polarization [13] of the digital and health industries. Although digital health is still in its nascent stages [14], today’s youth are technology natives [15], making the increasing transition to the digital delivery of health care inevitable. An improved social framework for design collaboration is critical for improving outcomes to facilitate better adoption, acceptance, and sustained use of DHIs [16]. Moving away from the tug-of-war between health care and digital design and instead toward a collaborative coproduction of digital health would represent a paradigm shift toward a truly transdisciplinary field [16]. In essence, the dualism of competing interests (digital and health) must give way to holistic design approaches that account for the constraints and affordances of health care and digital design collectively.

To better understand digital health design approaches, we reviewed 120 papers in the digital health space spanning qualitative, mixed methods, and case studies that present various co-design approaches to DHIs. We identified 5 overarching design approaches, examining the nuances in approaches and recommending their suitability for various industry use-case scenarios. This spanned traditional user-centered design (UCD) approaches to nuanced person, human, and patient-centered design approaches that seek to tailor various health care use-case scenarios. In doing so, we sought to examine the nuances in the approaches and recommend their suitability for various industry use-case scenarios. We hope that this research contributes toward the transdisciplinary evolution of digital health.

With the future of health care delivery becoming increasingly digital—more independent, self-managed interventions are being facilitated. Our research identifies the complexity of the sociotechnical arena that is digital health, one where 2 worlds with 2 different approaches are merging together to deliver health care. By examining the history, evolution, advantages, and challenges of industry implementation, we sought to identify growing pains in a hybrid industry that is in its adolescence.

## Methods

### Review Framework

This narrative literature review provides a descriptive and contextual detail on emerging digital health design approaches. Performed between September 2020 and April 2021, it maps a broad range of research domains, topics, strategies, experiments, and observations. The flexibility of the review approach is important, considering the broad stakeholder base in digital health from quantitative to qualitative research (inclusive of various perspectives: health care, engineering, computer science, human-computer interaction, psychology, design, etc). In this light, a narrative review allowed us to incorporate a broad spectrum of studies (and viewpoints) that would be difficult to facilitate in a systematic review. The broad range of findings were analyzed, compared, and contrasted for the synthesis and contextualization of key findings.

### Search Strategy

A literature search was conducted using the following electronic databases: MEDLINE, PsycINFO, CINAHL, Scopus, and Web of Science. In addition, the searches were supplemented with findings from Google Scholar and JMIR. Key search terms included “digital health design,” “mHealth design,” “e-Health design,” “agile health,” and “agile healthcare” in various combinations.

### Eligibility Criteria

The search strategy resulted in title and abstract retrieval based on any of the following inclusion criteria: (1) the study described an evaluation or protocol for a DHI; (2) the study described or evaluated an observational study (ie, design workshop); (3) the study detailed a case study (single or multiple) involved a digital health design approach; (4) the study proposed or described a digital health design methodology or methods; (5) the study provided a viewpoint or commentary on digital health design (ie, framework, policy, design, or evaluation); (6) the study was published between September 1, 2015, and December 31, 2020; and (7) the study was published in English. Studies were excluded if they (1) involved single user or patient studies; (2) focused solely on technical validation (ie, automated testing); and (3) did not discuss or evaluate end-user involvement in the study (ie, in design, development, usability, framework, or strategy).

### Data Collection and Analysis

**Figure 1:**
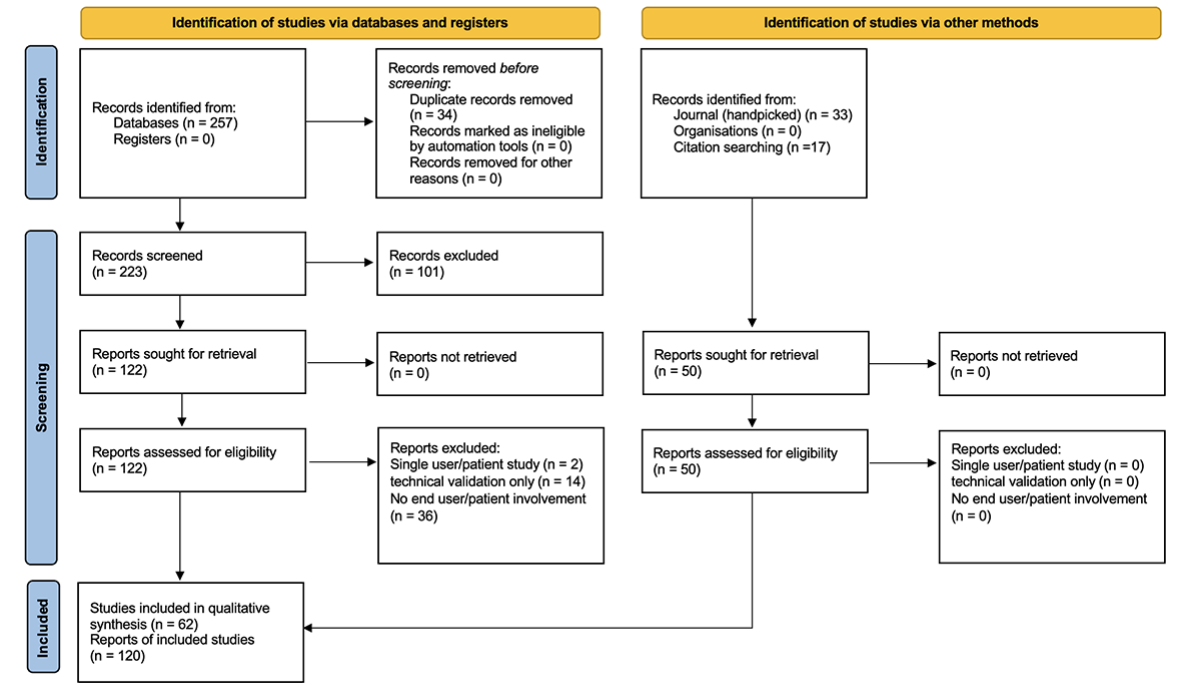
PRISMA (Preferred Reporting Items for Systematic Reviews and Meta-Analyses) flow diagram.

The first author completed the searches with assistance from librarians at Simon Fraser University, who reviewed search strategies, reference lists, and the relevancy of results. The identified titles and abstracts were downloaded and organized using Paperpile (Paperpile LLC). The first author independently screened all titles and abstracts against the defined eligibility criteria. After title and abstract reviews, full papers were assessed for inclusion by all authors. Considering the broad spectrum of design approaches and use-case scenarios in the emerging digital health space, studies from a wide variety of journals and sector vantage points were included. This included experimental, observational, methodological, case studies, and commentary-based studies. From this investigation, we extracted the 5 most prominent, most frequently occurring design approaches for analysis. A total of 120 studies were analyzed in full text. After full-text analysis, 62 studies that satisfied the inclusion criteria were included in this study. A visual overview of the PRISMA (Preferred Reporting Items for Systematic Reviews and Meta-Analyses) flow diagram is presented in Figure 1. Additionally, prominent findings from the literature review are presented in Table 1.

**Table 1:** Foundational publications on digital health design approaches.

Study			Type		Focus		Summary
**Digital health intervention design**							
	Blandford et al [13], 2018	Commentary		Interdisciplinary research		Seven lessons on the multidisciplinary approach of health and HCI^a^ to identify user needs and co-design interventions. The rupture between formative evaluation (HCI) and summative evaluation (health) is ever present in the cultures, values, and design assumptions presented.	
	Shaw et al [17], 2018	Commentary		Implementation		The potential impact of a service-design approach for improving the triple aim of health services (enhance patient experience, improve health outcomes, and reduce costs). A perspective on shifting from traditional implementation to an interactive cycle of value proposition design.	
	Thabrew et al [18], 2018	Review		Children and young adults		A summary of the core principles of agile co-design (the collective creativity of all stakeholders throughout a design project) in eHealth interventions for children and young people.	
	Hekler et al, 2016 [19]	Commentary		Behavior intervention		An adaptation of agile science principles for real-world behavior change in health care. Adapting and adjusting evidence-based research to specific individuals and contexts.	
	Birnbaum et al [20], 2015	Commentary		Patient engagement		Digital health intervention design has shifted away from top-down implementation models to seeking to bridge the gap between health products and patient needs. A discussion on the evolution of UCD^b^ to (PCD^c^) and (PLD^d^) as a health-centric response to this challenge.	
	Poole [9], 2013	Commentary		Interdisciplinary research		A call for interdisciplinary cooperation among technologists, health researchers, and HCI experts to address user acceptance and adoption in mobile health. The research highlights the barriers to successful collaboration.	
**User-centered design**							
	Duque et al [21], 2019	Review		Older adults		A systematic review (2013-2018) of UCD approaches with older adults, including discussion on the challenges in better involving older patients in a UCD process.	
	Wysocki et al [22], 2018	Observational (design process)		Parent (caretaker)		A mixed methods study of parents of children aged <6 years with a chronic disease. The research describes the UCD process, illustrates the reach of crowdsourcing for design inputs, and summarizes the results of a randomized controlled trial.	
	Vilardaga et al [23], 2018	Observational (design process)		Mental health		A stage-by-stage walk-through of applying a UCD process in the design of a mobile health smoking cessation app; from the rationale, ideation, prototyping, design, and user research to the final feature set. Learnings are systematically reported from each stage.	
	Azimi et al [24], 2017	Review		Older adults		A discussion on the Internet of Things and its propensity to assist care for older adults and remote monitoring. An exploration of current UCD approaches in care for older adults is examined along with recommendations for future development.	
	Lyles et al [25], 2016	Observational (design process)		Primary care		An exploration of a UCD approach including patients, providers, and health stakeholders to improve primary care tools in iterative stages.	
	Lyon and Koerner [26], 2016	Commentary		Implementation		A report on using a UCD approach for psychosocial interventions as a supporting exploratory approach to evidence-based treatment. The “fail fast” mantra of agile development is weighed against empirical approaches in traditional health care.	
	Curtis et al [27], 2015	Observational (design process)		Caretaker		A blended approach of the behavior change wheel, UCD, and commercial approaches to systematically design a childhood weight management app. Parents were primary stakeholders through the process.	
**Person-based design**							
	Devlin et al [3], 2016	Review		Implementation		Examining the implementation lessons from a large-scale deployment of a person-centered assisted living program. The challenges to work with heterogeneous groups, the resilience to break through barriers, the tensions in co-design processes, and the inherent market pressures to deliver products are all explored.	
	Yardley et al [28], 2015	Commentary		Methodology		3 illustrations of how person-based design can be used to improve acceptability and feasibility in the formative design stages.	
	Yardley et al [28], 2015	Feasibility study		Behavior intervention		An understanding of the person-based design approach through the initial stage of planning, feasibility testing and implementation, and the second stage of identifying guiding principles to inspire and inform more context-specific behavioral issues. The perspectives of the people who use the solution are central, beyond the typical user-based analysis and validation.	
**Human-centered design**							
	Wheelock et al [29], 2020	Commentary		Methodology		An overview of (HCD’s^e^) overarching philosophy and its methods and practical implementation in health care. The analysis discusses the challenges to build trust within a complex stakeholder group and a call for better co-design methods to navigate this challenge.	
	Chancellor et al [30], 2019	Review		Mental health		A systematic literature review of human-centered machine learning exploring the human in HCD. The study resulted in 5 key findings on how the human is understood: (the specific) disorder, social media, the scientific, the data or machine learning, and the person.	
	Ragouzeos et al [31], 2019	Observational (design process)		Patients		An experiment to observe the collaboration of patients, designers, IT experts, and clinicians in an HCD process to prototype a rheumatoid-arthritis intervention.	
	Holeman and Kane [32], 2019	Review		Implementation		A contextualization of HCD for global health equity, and the unique offerings of HCD over traditional health care approaches to research and innovation. The research tracks over 70 HCD driven digital health initiatives.	
	Mummah et al [33], 2016	Framework		Implementation		IDEAS (integrate, design, assess, share), a framework strategy to design, develop and evaluate digital interventions and health behavior change incorporating a wide swathe of human-centered factors.	
	Harte et al [7], 2017	Framework		Implementation		A 3-phase methodology that blends use-case scenario, expert usability analysis and user testing in a *connected health* format that is iterative, seeking to improve human factors in collaboration.	
**Patient-centered design**							
	Grisot et al [34], 2020	Case study		Implementation		An examination of designing for recombinability in health care. A total of 2 case studies are studied to better understand the blending of patient-centered approaches into health care design.	
	Boissy [35], 2020	Viewpoint		Implementation		A proposal for *operationalized empathy*, redesigning patient experience measurement and developing organizational readiness for patient-centeredness.	
	Espay et al [36], 2019	Review		Implementation		A discussion on how to road map a hybridized patient-centered and clinical outcome in the digital space for Parkinson disease.	
	Carter et al [37], 2018	Viewpoint		Implementation		A conceptualization of “clinician-innovators”: the merging of technology-enabled innovation and patient-centered care to bridge the implementation gap in digital health.	
	Van den Bulck et al [38], 2018	Cross-sectional study		Health informatics		An implementation road map for patient-centered digital outcome measures that considers patients characteristics, benefit-to-burden ratio, integration actualization and regulatory approval within the digital health system.	
	Tang et al [39], 2016	Viewpoint		Implementation		A discussion on the effectiveness of patient-centered information systems considering social and economic factors as well as disparity in multisector health outcomes.	
**Patient-led design**							
	Kempner and Bailey [40], 2019	Case study		Patient engagement		A case study examining 2 websites on collectivizing self-experimentation and crowdsourcing in patient-led approaches.	
	Stolk-Vos et al [41], 2018	Feasibility study		Patient engagement		Feasibility study for the design of a patient-led hospital checklist to promote patient engagement and broader collaboration with health care professionals.	
	Leese et al [42], 2017	Case study		Patient engagement		A case study walk-through on a patient-led collaboration that discusses the practical, ethical, and sector disconnect issues in negotiating a patient-led approach.	

^a^HCI: human-computer interaction.

^b^UCD: user-centered design.

^c^PCD: patient-centered design.

^d^PLD: patient-led design.

^e^HCD: human-centered design.

## Results

What follows is a narrative synthesis of the historical context of the health care and digital industries, respectively, and the subsequent emergence of prominent digital health design approaches are discussed, including origins, advantages, disadvantages, challenges, potential use cases, and nuances.

### Health Industry

The health industry’s *do no harm* [43] approach centers intervention design on systematicity, transparency, and rigor. Methods must be provable and reproducible [13]. This approach is built upon a pharmacological intervention mindset that posits randomized control trails (RCTs) as the gold standard for health intervention evaluation. The health outcome of an intervention is the key metric of concern [9].

Ironically, the rigorousness of clinical evaluation is also what challenges its implementability in digital health. RCTs tend to take many years to present outcomes, whereas digital production cycles spin iteratively in a matter of months. This mismatch in pace [14] challenges the boundaries of an evaluation framework. Clinical studies are also not designed to account for usability testing that could evaluate patient safety [4]. The nuances that affect patient uptake, the UX that fuses the sociotechnical domain, are often not considered in the health industry approach. A key challenge to the waterfall model [13] of systematic research in the health domain is the rinse-and-repeat rapidity of iterative design in the digital industry.

### Digital Industry

The digital industry’s *fail fast, fail often* mantra [43] is premised on rapidity, iteration, and an overall understanding that the solution will emerge organically. This approach is rooted in the belief that it is impossible to fully understand the user’s needs ahead of time [13]. Therefore, rather than front-loading research, it is evenly distributed and prioritized during an agile evolution of ideation, prototyping, and testing alongside user participation and evaluation. This approach lends itself to innovative projects by reducing costs [26] and interacting with potential users [33] early and often. Broadly interdisciplinary, the digital industry often prioritizes a qualitative approach with flat management teams that consider human factors, computer science, information systems, psychology, sociology, and visual design [9].

Nonetheless, the digital industry’s swift production cycles are incompatible with paradigmatic long-term health evaluation [13]. Although the digital industry can succeed in bringing a product to market within a short time frame, its rapidity and lack of rigor cannot satisfy the clinical depth of research of health validation, one that is inclusive of a more long-term, data-driven quantitative analysis.

### Emergence of Digital Health

#### A Clash of Cultures

It is within this clash of cultures that the digital health industry is rapidly emerging. Despite overlap in interests [9] and a mutual desire to improve health outcomes, the industry has experienced the growing pains of harmonizing what a design approach entails to be both *digital* and *health* collectively. Digital professionals often view health research as too time-consuming and *straight-laced*, whereas health professionals often view digital research as *scruffy* and unreliable [13]. The digital push for rapid innovative solutions is pulled back by the desire for long-term safety and efficacy in health care. The implementation gap can be bridged by a more transdisciplinary approach that binds together health care operations, clinical informatics, and digital design in a more fluid process [37].

To do this, optimized design approaches are needed that position cocreation as a fundamental pillar of the digital health value proposition [17]. For adoption, acceptance, and sustained use [16] of DHIs to be improved, a paradigmatic shift toward a participatory silo-less domain is required. A transformational [37] approach that requires digital professionals to weave health requirements into the affordances and constraints of intervention design, and for health professionals to embrace design thinking [44], can better orient DHI design around human factors and user experiences. Product design and health care design can no longer be demarcated. The bidirectional relationship between patient and health care service is omnipresent in today’s digital world [45].

While curating a DHI, there are a number of key areas of conflict to overcome.

#### Design

While the digital industry considers a user-centric process that defines an intervention by the needs of the target user in a narrow, fast-paced goal orientation, the health industry is expert-driven, and considers a broader, more complex design framework that begins long before software development and extends long after its rollout [13].

#### Evaluation

While the digital industry focuses on UX in the form of qualitative feedback, such as user testing and analysis, health experts look to evaluate the effects and impact of an intervention as a successful or unsuccessful health outcome [13]. The former method can occur over a short period with a limited sample size, whereas the latter is expansive, is detailed, and can occur over years.

#### Validation

The digital industry values technical validation to ascertain usability and user validation to ascertain positive UX and potential uptake. The health industry conducts clinical validation to understand whether the intervention provides efficacy for a condition-specific content [43]. In addition, it conducts system validation, which considers a wider scope of patients, providers, and the health care system as a larger network of health care delivery [43], a broader marker of the overall success of the health intervention.

#### Implementation

The digital industry understands this to be the final layer in the product timeline, the release, and handoff of a digital health product. In the health industry, implementation is a complex systematic process of strategic planning and expert consultation, guided by clinical governance. It is not an end point but an ongoing research into health care efficacy. A digital health product would be but one factor of the whole implementation [13].

The variance in designing, evaluating, validating, and implementing interventions forms the core problem space for digital health stakeholders. To reduce this complexity and improve intervention quality and uptake, a number of design approaches mediate the digital health space. In our review of the following 5 design approaches (summarized in Table 2), we weigh their strengths and weaknesses and evaluate their challenges toward industry implementation.

**Table 2:** A comparison of the advantages and challenges of 5 key design approaches.

Design approach	Advantages	Challenges
UCD^a^	A large research community to draw upon with broad use in human-computer interaction and related fields. User-validated process directly addresses uptake concerns in DHIs. Broad approach is adaptable to all categories of DHIs.	Defining the end-user in health care interventions is difficult because of the complex collaboration of stakeholders in DHI^b^ facilitation (ie, clinicians, caregivers, and patients) who may all be end-users of the DHI. Aligning preferences to patient end-users may conflict with health policy based on expert-led evidence-based practices. Largely qualitative feedback often represents a small sample size that opposes the rigors of traditional longitudinal health metrics.
PBD^c^	Psychoanalytical approach that contextualizes improved well-being as a design outcome is well suited to behavior changing DHIs. Empathetically guided “sensitive design” process broadens stakeholder focus beyond active users to also include passive users and collaborators in the DHI as a whole person network approach.	Behavior change metrics from PBDs may not be transferable to other types of DHI designs. Psychoanalytic “sensitive design” may create an expert-led barrier to entry for other collaborators in the DHI (developers, designers, etc) and add scope.
HCD^d^	Highly adopted and International Organization for Standardization–recognized approach for system design, already has health care provider backing (Mayo Clinic, Kaiser Permanente). Combining approaches of user-centered design, human-computer research, anthropology, and sociology under the banner of “social innovation” has broad appeal to unite a wide swathe of DHI collaborators.	An “umbrella term” that approaches design ethos and policy framework from many fields of research, there is a lack of unified guidelines, thus there is a need for a demonstrable lightweight framework for DHI design and facilitation. Project scope is challenged by the breadth of collaborators (patients, clinicians, designers, developers, and academics) which may expand timelines in a fast-paced design environment.
PCD^e^	Pivoting focus from user to patient (commercial to health care) creates better alignment with health care infrastructure policy, allowing for better buy-in from health care stakeholders. Empowering patients to take leadership of their health care management is a leading metric in DHI retention and advocacy (particularly in wearables and sensor-based DHIs).	Positioning patients as primary stakeholders (or as experts) oversimplifies the complexity of health safety and clinical efficacy guidelines and may lead to undesired patient outcomes. Crowdsourcing DHI preferences may lead to misdiagnosis by popular convention, democratized data sets will still need to be weighed against medical best practices.
PLD^f^	Self-tracking, self-analysis PLD approach is positioned well for today’s emerging personalized health care marketplace. Machine learning–backed “citizen science” approach offers large quantitative data sets for better triangulation of patient preferences.	Patient-led approach may lack the holisticity of HCD or PBD and the safety and efficacy of traditional health care methods, this may limit the focus to preferences rather than clinical health outcomes Scalability is questionable because of limited stakeholder base (lack of consensus) and self-experimentation approach (lack of clinical validation).

^a^UCD: user-centered design.

^b^DHI: digital health intervention.

^c^PBD: person-based design.

^d^HCD: human-centered design.

^e^PCD: patient-centered design.

^f^PLD: patient-led design.

### Design Approaches

#### User-Centered Design: 12 Studies

UCD is a qualitative design framework with roots in the human-computer interaction community dating back to the early 1980s [21]. It builds validation and satisfaction around the end user [13] by understanding personas, preferences, and environments through an iterative design approach. The goal is to output purposeful design, with the understanding that the intuition of experts alone is often insufficient for user validation [9].

Therefore, UCD focuses on the routine everyday needs of users and their circumstances, resulting in a design philosophy that guides the development phase iteratively [13]. By appealing to the conscience of users, situating them as primary stakeholders, and involving them in the design process, usability can be proposed, tested, and verified in a cyclical process, prioritizing the needs of the users in real-world situations [25]. A result of the UCD process is to determine why a design in a given environment with certain constraints and affordances is successful in one instance with a given set of users but unsuccessful in another, and how to mediate these design challenges [9].

The shift to a UCD approach in health care converts the traditional patient-physician relationships to a more reciprocal collaborative space, particularly in the development of self-monitoring and self-management apps [1]. The implementation of UCD approaches in health care is very much in its infancy [24] but holds the propensity for greater patient empowerment. Involving users in ideation and using a visual storytelling approach that involves workshops and gamification may invoke rich emotional feedback that helps feed health application design. These metrics are mutually important for the advancement of broader scientific research on efficacy, usability, and safety [23].

Positing the user as the primary stakeholder is not without limitations. Traditional approaches to health intervention design that are rooted in evidence-based practices or theory-based principles of change [23] may oppose the user-centricity of intervention design, seeing that user validation is not the sole desired output of a health intervention. In health care, a variety of expert viewpoints exist specific to the type of intervention. This often involves physicians, health experts, government, nonprofits, and other stakeholders who are part of a holistic health intervention. Positioning the user as the expert [13] may challenge long-standing traditions of clinical expertise in the health industry.

An example of this conflict is a *stop smoking* application that offers users advice and notifications on how to quit smoking. In a UCD approach, the input, ideas, and feelings of users would be central to the application design. Research conducted by Cheong et al [46] showed that smokers (users) widely believed that cutting down on cigarette use is the path to quitting smoking. Validated data from health experts showed the contrary, that stopping outright was statistically the most successful approach [46]. In this context, a challenge exists: academic research and expert analysis are not automatically factored into a UCD simply because neither may be end users.

Despite the disparity in approaches, the value of exploring UCD in digital health is driven by the inexorable link between technology and health care delivery [27] in the form of mobile health (mHealth). UCD is being used to facilitate lifestyle and self-management of chronic conditions such as diabetes [22]. The mutual interest of the physician and patient in the metrics produced by users exemplifies the rich potential of UCD in health care, one where agency is inevitably shifting to the end user [5]. In both the collaborative ideation phase and in postdeployment observation, the UCD identifies both challenges and trends in end-user behavior.

In summary, UCD is one approach that helps shift the evaluation of DHIs from postrelease [25] to the design phase, with an eye toward pivoting intervention designs according to user feedback. Rather than front-loading research and delaying evaluation, research and evaluation are fluid processes happening throughout the life of the design. It is hoped that, in doing so, design flaws are reduced or eliminated, and simultaneously, user engagement is increased. Despite the rupture a UCD approach may cause to traditional health care approaches, a key buy-in is the potential for scientific discovery through the multidisciplinary nature of design ideation. Improved contextual design, particularly in complex health interventions, can address both efficacy and cost concerns [26]. In seeking to smoothen the edges of UCD in health care, a number of emerging approaches have been developed that center on human-, person-, or patient-centered design (PCD). These nuances offer a tailored approach to traditional UCD.

#### Person-Based Design (4 Studies)

Person-based design (PBD) is a new space [3] that seeks to humanize the design approach, neither framing participants as users or patients [8] but more generally as the people who use the intervention [13]. Building on UCD, it layers on mixed methods qualitative research in the form of behavioral theory and analysis [28]. Building the intervention around the stages of planning, optimization, and implementation, it seeks to enhance feasibility and acceptability through an intervention design that is sensitive to the lives of the people who use them. A broader psychoanalytic method, self-determination theory [8] is cited as a reason to expect improved uptake when people feel a sense of *acted user agency* in the design process. It is also understood from this approach that a variety of people contribute to a holistic solution as stakeholders, not just users, patients, or experts specifically [8]. PBD aims to help intervention designers understand how people (patients, health care workers, family members, etc) experience and implement a given intervention; these nuances create unique insights for the design process, beyond the user or patient perspective alone [8].

PBD separates itself from user-centered and patient-oriented designs by focusing on motivation, enjoyment, informativeness, and convincingness. This approach is more empathetically rooted than traditional UCD metrics built around usability, acceptability, and user satisfaction [8]. Enhancing the well-being of the person, rather than validating the experience of the user, is the differentiator. An example of the advantageousness of PBD is in the contextualization of sensor data interpretation. From a data-centric viewpoint, restaurant app users were tracked to see when they were near fast food restaurants, and then prompted with a notification. The context sensing at play would seem logical from a mapping viewpoint. However, in a PBD study, it was found that users were skeptical or annoyed about notifications raising trust concerns. This psychoanalytic approach contextualizes emotionless data points that do not speak to the feelings and behaviors of people [8].

While UCD maps a user’s knowledge and skills, validating them on a basis of user satisfaction, PBD uses health psychology to validate a person’s responses wholly [8]. Similar to UCD, PBD also faces the challenge of contrasting research methods with traditional health approaches [13]. PBD approaches often form an iterative workshop base [3] similar to many agile UCD practices. Person-based advocates position it as a complement to existing theory-based and evidence-based approaches [8], although being focused on behavior change [8], questions exist as to how broadly or narrowly it can be used in health care [8]. Therefore, a key consideration is how to blend the PBD framework into the industry-practiced agile ideation and prototyping cycle, leveraging the advantages of both methods.

#### Human-Centered Design (8 Studies)

Human-centered design (HCD) has evolved over the last 3 decades from human factors, human-computer interaction, anthropology, sociology, and UCD. It is an interdisciplinary approach to create social innovation in the health domain [32]. HCD is recognized by the International Organization for Standardization as a standard for interactive system design [33]. It has been adapted by the Mayo Clinic Center for Innovation and by global health care provider Kaiser Permanente [29]. The “human” in HCD signifies a broader social and organizational construct, prioritizing the aspirations and experiences of people holistically [30]. The foundational layer of the HCD is empathy. Before turning to traditional UCD phases, such as defining, ideating, prototyping, and testing, empathy is used to understand the underlying barriers, conflicts, and root causes related to pain points [37]. Although UCD may pivot design based on end-user pain points, HCD first asks [33], “Why is there a pain point and where did it come from?” In this regard, it may align more suitably with mainstream behavioral health practices [33], potentially increasing buy-in and reducing the rigidity of porting tech industry UCD practices into health care.

HCD seeks to create a deeper, more meaningful involvement of end users, observing and interviewing patients, clinicians, and various members of the health care team. The holistic approach seeks out a “right time, right place” method of capturing the collective experiences of the human who is mediated by the intervention [31]. This real-time intervention adaptation [36] binds together the collective brain of diverse stakeholders around the human. The focus on HCD shifts the lens from building technologies to building a framework to interpret and resolve complex DHIs at their core [32]. As an umbrella term [32], it can be difficult to define explicitly; nonetheless, it differentiates itself from UCD by pre-emptively aligning the technological intervention with people’s values, concerns, and day-to-day needs. This includes documenting the participation of potential users, supporting cooperation with them, and augmenting human skills in the design approach [32]. By collaborating to specify the context of the intervention [29] from a human and health behavior context, there is an element of design before the (digital) design. The HCD approach provides empirical evidence that may satisfy both clinical concerns over UCD brushing over holistic health research and designer concerns over articulating purposeful design for end users. HCD may serve as a bridge between health care and digital approaches, fostering greater trust among stakeholders [29].

Among the challenges for HCD to overcome is the fact that, unlike most health care processes, it is not systematic [33] with clear guidelines. In some circles, it is seen as a buzzword [32] with vague demarcation points among design, development, engineering, and health care. Arguably, this is exactly the juxtaposition desired to drag out empathetic insights for a more holistic design approach. Research conducted from a singular, siloed vantage point may struggle to provide the wholeness of HCD. In contrast, HCD provides a voice to humans [36] who will depend on the given DHI, through the broader lens of the collective interpretation of a digital health team. Another challenge is that HCD can sometimes be viewed as superficial [32] and impractical. Gathering patients, clinicians, designers, developers, and academics under one tent is fine in theory but difficult to implement in reality. This is further complicated by the desire for pace (from the digital side of the room) [7] and the desire to move slowly and cautiously (from the health side of the room). Development teams may not be eager to add scope before the scope in the form of empathetic discovery sessions [32]; physicians may not see the value in various theoretical approaches to medical or pharmaceutical interventions. HCD practitioners will argue that no amount of expertise built upon abstract assumptions substitutes the deep intuitive data points from observing and collaborating with all stakeholders [32], from patients to experts, in the wild. HCD may not offer the fixity of a systematic health protocol [32], but instead it offers a theoretical framework for the interpretation of complex DHIs free of bias that may skew the intervention design away from the needs of the humans who use them. Moving forward, the ability to scale up an HCD for a more policy-driven rollout will be challenging [32]. Considering that HCD is vastly open to interpretation, the continued cross-functionality of digital health teams will be pivotal for developing emerging rubrics.

#### Patient-Centered Design (12 Studies)

PCD nuances HCD, specifically pivoting to the needs of the patient. The British National Health Service’s motto, “no decision about me, without me” emphasizes the need for patient-centered shared decision-making [38]. In 2001, the Institute of Medicine authored a report calling for 6 improvements to health care delivery, among which was a patient-centered approach that is responsible for individual patient needs and values, guiding clinical decisions [35]. By 2006, the Picker Institute issued a guide that was built upon the Institute of Medicine report, citing the need for better education and shared knowledge, more collaborative approaches, and more consideration of patient needs and preferences [35]. This backdrop coincides with the emergence of DHIs over the past 15 years that can enhance patient engagement, but with that is the fear that “tools are not enough” [38], that the needs of the patient should guide the design approach. The concept being that patient validation maximizes acceptability and usability [47].

The PCD approach targets patient-facing technologies such as personal health records, patient portals, and mHealth apps [39]. In this regard, it hopes to provide a digital pathway to the triple aim in health care of improving patient experience, reducing costs, and improving health [17]. PCD seeks the patient to take leadership roles in their care, rather than being empowered by professionals [48], through qualitative patient perspective workshops that are interesting and enjoyable [47]. It pivots the UCD approach to user needs and wants, reframing them as patient needs centered on achieving therapeutic benefits and patient wants being intervention designs that guide retention [47]. In doing so, it shifts traditional industry UCD approaches from consumer oriented to patient focused. This logic aligns better with health care infrastructure and policy [34]. Borrowing from HCD, PCD operationalizes patient empathy [35], seeking out metrics that show a patient trajectory moving from passive to active participation [34], a key indicator of more knowledgeable, more empowered patients.

PCD is proving influential in wearable, sensory-based technologies that quantify the self. An explosion in digital health technologies (DHTs) that are lifestyle interventions—self-tracking, self-experimenting in diet, exercise, and sleep [49]—has demonstrated the valuation of more human or patient-centered interventions. By their nature, wearables provide a bilateral relationship between the end-user and the health care industry. This real-time data demonstrates not only the needs and wants of users, but also their behavioral interaction with DHIs. In another example, Johns Hopkins Hospital created an app for discharge that shifts from paper to digital, reengineering, and expediting the process, putting the agency in the hands of the patient [10]. This process still requires constraints; however, the positioning of the patient in a proactive role accentuates the National Health Service's call for more engaged patients [34].

Although patient empowerment and personal agency are undoubtedly factors in improved design and uptake, centering the patient as a primary stakeholder is not without challenges. A patient may desire a DHI design that is discordant with proven clinical efficacy [31]. For example, an app that manages the dispensary of medications may need clinical checks and balances to avoid side effects or abuse. This may not fit the preferences of the patients centering on the design. Overconfidence or social crowdsourcing of ideas may incorrectly influence patient mindsets. The diagnostic accuracy of PCD can be easily questioned. In this regard, it is difficult to imagine this as a standalone design approach [36]. In addition, PCD as a broad approach can be seen as an oversimplification of the complex and intricate domains of health care [34]. Disease management and urgent care often have very specific and time-sensitive approaches that cannot be opened to popular opinions. Also, the qualitative, rapid approach to PCD data points is difficult to correlate with gold standard RCT data sets that are quantitatively vast [36]. With smaller data sets, PCD approaches often bring into question who the patient is, how diverse the demographic is, and why they were chosen [36]. This is not to say that PCD is not impactful but rather that it has a particular scope and context to better understand patient thoughts and preferences for intervention design [36]. This scope is challenged when patients contradict medical best practices. Regardless of its influence in clinical decisions, PCD provides insights into patient preference and behavior that other design approaches may not uncover.

#### Patient-Led Design (5 Studies)

Furthering the patient-centric approach is patient-led design (PLD), a design approach that considers patients as partners [42]. Taking an example from the web 2.0 phenomena of prosumerist crowdsourcing, the approach understands that patients themselves are proactively taking the lead in curating their own health care through digital means, a health care 2.0 [40]. This approach resonates with the transition from “sick care” to health care, one where personalized health care is built around the patient’s self-tracking and self-analysis [48]. Preliminary research, ideation, and design all function within the discovery of patient-led initiatives to equip, enable, and empower patients [20].

A key advantage of PLD is the ability to rapidly garner results from large samples of the population [40]. This crowdsourcing approach, coupled with advancements in machine learning, can provide rich data sets, a quantitative means to triangulate results in other qualitative studies in design approaches (HCD, UCD, etc). In addition, the self-experimentation of patients that occurs during the curation of DHIs leverages a form of citizen science such that innovative treatments may evolve from the process [40]. Similar to PCD, buy-in is easier to attain when patients are treated as the primary stakeholders. This approach can be particularly useful for startups and low-budget projects seeking new and innovative DHIs.

Similar to PCD, PLD is challenged by the shifting balance of power dynamics between patients and clinicians [41]. As more agency is given to the patient in the design and curation of an intervention, less is given to the traditional health care base, raising questions regarding safety and efficacy. Weighing it against HCD, questions remain about the holisticity of the approach, one that could benefit from a richer group of stakeholders in the formation of digital health solutions [40]. Self-experimentation juxtaposes the standardization of medical treatments. Health literacy is not a prerequisite for PLD.

In counterbalancing the constraints and affordances of PLD, it can be argued that more patient participation can lead to increased health literacy, greater understanding of safety, and a shared responsibility in balancing power [41], something of interest to patients and clinicians alike. It is hoped that PLD reveals new types of patient engagement in the highly participatory digital space [42]. Considering that Reddit-like digital coffeehouses are only likely to increase with augmented reality and machine learning technologies, the goal of leveraging these data points and mixing them with qualitative findings opens up an avenue for more robust research methods. Increasingly, DHTs will provide more tailored interventions that develop diverse data points around the patient’s feedback. The physician of tomorrow may increasingly be oneself [48], mediated by algorithmic deep learning. Ignoring this transition would be unwise. Nonetheless, understanding the context of when PLD is resourceful versus when it may be harmful within a DHI design remains a question moving forward.

### The Golden Thread of Collaboration: Participatory Design, Co-design, and Cocreation

Each of the aforementioned design approaches shares the general values of participatory design, co-design, or cocreation. This approach is supported by the UK National Institute for Health Research [50], with the foundation being that the intersection of various sciences and learned experiences harmonizes DHIs [3] in the form of *social innovation* [51]. The cultural shift [52] to more autonomous, pervasive [53] DHTs has enhanced value cocreation in digital health as a strategy of increased patient involvement, reduced costs [54], and better uptake. Participatory co-design methods mesh industry toolkits and workshops with a wider swath of human- and patient-centered strategies [55]. This involves collaborating with end users and a diverse array of professionals in preideation research, ideation, prototyping, testing, and postlaunch retrospectives, synthesizing the understanding of health, technology, and design experts, anchored upon the insights of the user [53,56]. This approach has been referred to as a *golden thread* that runs through every stage of the intervention, looking through the lens of the target user throughout [18,56].

This equal partnership approach [57] studies what the end user says about the DHI, does with the DHI, and makes from the DHI [18]. The nuances in each of the aforementioned design approaches shift the focus from user to human to patient, each pivoting the mission statement slightly in search of new and groundbreaking approaches. However, they each share the essence of the co-design ethics of mutual learning, democratization of power relations through shared ownership, and use tools and techniques to facilitate better collaboration [58]. Designing innovative health solutions *with* and not *for* end users is the desired outcome [43].

In doing so, closing the gap among clinical, technical, and design perspectives is oriented around not *what is* but what *could be* [51]. This can be an uncomfortable process in health care as it questions traditional practices and favors a new, broader body of knowledge [57,59]. While it may increase the sense of belonging [60] in patients, it may also decrease the sense of worth in clinicians. Nonetheless, co-design seeks to shift the voice [13] of DHIs to an interdisciplinary domain that is more reflective of the digital ecosphere today.

There are a number of key challenges in this shift. Moving digital health design approaches from theoretical to practical involves resolving the cultural differences between health care and complementary domains involved in DHIs. Nonlinear, more agile pathways to DHI design need to be embraced. Cocreation methods as a project valuation are not widely understood in health care [61]. Cocreation rethinks health care delivery that impacts both the macro and micro level of the health ecosystem [61], a top-to-bottom cultural change that understands the shifting agency of increasingly digital health care facilitation. This is also difficult in practice because of the layered levels of bureaucratic governance, from regional to federal to international regulation [15], each having its own perspectives, priorities, and ethics. The fact that there are so many variations in how to deliver DHIs further complicates upstream changes to health care policy. Becoming comfortable with the uncomfortable [57] is part of the adolescence of digital health.

## Discussion

### The Promise of Digital Health

Digital health is emerging as an industry that gives promise to a more personalized health care experience. The demand for health care apps doubled between 2011 and 2015, reaching 165,000 apps [1]. In response, mHealth investment grew from US $4.4 billion to US $6 billion between 2016 and 2017 alone [43]. The digitization of health care delivery is increasing the autonomy of health care users. With this, a paradigm shift is emerging, wherein the agency of users is rivaling traditional health care practice that is primarily expert-based. There is a wider acceptance of pivoting the intervention design toward the user, person, patient, or human, part and parcel because of an emerging landscape of digital natives. Clinical professional assessment is becoming increasingly supplemented by self-analysis and self-management apps that shift agency toward the health care user. This is creating greater access with more robust data points, which curates personalized real-time data. This contributes to a faster, more intuitive health care delivery. Emerging design approaches are seeking to port digital and design best practices into health care solutions. Simultaneously, the rigors of health care safety and efficacy are in need of being compressed into digital delivery timelines. This dichotomy has created friction on how design, evaluation, and implementation are understood from the digital and health care sides of the room.

Among the key challenges identified in this research is the disparity between intervention design in traditional health care and digital settings. The hybrid ecosystem that is digital health faces a multiplicity of design approaches and countless use-case scenarios. These approaches have exposed a silo disconnect among various stakeholders and methodological differences in intervention design. Despite each nuance in the design approach shifting the vantage point of the primary stakeholder, it is often unclear how these design approaches can be tailored for rapid app development while balancing the safety and rigor of health standards. Although a PCD may prove effective in large stakeholder projects such as mHealth self-tracking diet apps, a patient-led approach may have fewer constraints, allowing for more crowdsourced experimentation in the development of innovations in DHTs. In contrast, person- and human-based design may appeal to psychoanalytic interventions for depression and mood disorders. There are no hard demarcation points between the design approaches, as they borrow and overlap techniques under the broader umbrella of co-design. This lack of systematicity accentuates both the promise and the challenge of digital health. The implementation gap [37] is the space that is allowing new, collaborative approaches to emerge. It is also the flashpoint of methodological differences.

### Improving the Future of Digital Health Design

Looking to the future, reducing the polarization of the 2 cultures (digital and health) [13] is paramount. As digital health matures, interdisciplinary approaches can become transdisciplinary and free of sector boundaries such that digital and health are undemarcated. On the part of digital experts, a better understanding of distal outcomes from a health perspective would be enlightening. Similarly, health experts would do well to understand the value of proximal user research and rapid iteration. Health concepts, such as efficacy and safety, can become hybridized with digital concepts, such as UX and usability. For example, a “user efficacy” can blend clinical and design principles that target both effectiveness and positive experience. Safety and usability can blend health constraints with technical affordances.

In addition, there is the value proposition challenge of absorbing the additional cost of design infrastructure and digitally upskilling stakeholders [62] in a co-design environment. Owing to this study being focused on defining and critiquing design approaches, we have not addressed this *elephant in the room*. However, financial challenges exist to justify these design approaches as part of a business-as-usual approach. Further studies should be considered to weigh the unique value proposition of a given design approach for a given health care sector or use-case scenario. For digital health collaborators to reduce friction and pain points, focusing on a value proposition design that establishes a digital application of the triple aim in health care is important. Considering that a 2019 survey found that only 57% of patients felt that physicians acted in their best interest [29], the digital agency of health care users can only serve to improve trust and uptake. In doing so, a value chain can emerge that keeps stakeholders across multiple disciplines—clinicians, academics, designers, and developers—mutually invested in an approach that is transparent, is effective, and, most of all, creates true digital health affinity.

To address these challenges from a research perspective, we recommend the following three steps:

1. Triangulation of the common challenges that bleed through all design approaches to help distinguish overarching pain points in digital health. To that end, a systematic review focused on the key challenges in incorporating end users in the design of DHIs would be instrumental.
2. User studies that illustrate collaboration with industry partners to blend various design approaches into agile workflows would demonstrate pragmatic implementation in health care app development. This will help with proof of concept, providing real-world analysis and value proposition. It will also explore and resolve issues of practicality and scalability because of real-time industry constraints.
3. Case studies that involve digital experts spending time in health care environments to understand what efficacy and validation implies in a health care context. Similarly, a study of health experts who reach beyond consultancy, instead fully participating in agile development cycles, from ideation to product release, is needed to increase our understanding of purposeful design and user validation.

Although the hybridization of digital health may feel forced, the aforementioned steps may encourage a more organic and unified approach to design.

### Conclusions

The future of health care is becoming increasingly digital. The proliferation of artificial intelligence [45] in the form of machine and deep learning [30] offers limitless real-time [1] insights that promise to further fuse together digital and health into the mid-21st century. In its infancy, this interdependent relationship has been strained. The various approaches to manage differences in digital and health care design center around various forms of collaborative co-design. The goal of bringing together 2 vastly different industries (digital and health) under one umbrella is a complex challenge that is being explored using various design approaches. Although each of the studied approaches offers a nuanced take on how to create purposeful design in digital health, positing the challenge around the user, person, or patient shifts the vantage point of the primary stakeholder only slightly. Of greater concern is how to create a truly transdisciplinary environment in which a culture of digital health emerges that is less tribal and more agile, reducing the friction of competing interests. To accomplish this, a demonstrable value proposition that proves faster, better-quality, more efficient, and more user-empowered solutions is needed. In doing so, there is the potential for better buy-in from all stakeholders. Further research is needed to analyze the pragmatic and cost-effective demonstration of each design approach in a real-world context. Finally, piloting these design approaches within robust design teams that expand the usual array of project managers, designers, and developers to include clinicians and health experts—from ideation through to deployment—can lay the foundations of an emerging digital health culture, an ethos that balances the needs of health care and design equitably.

## Abbreviations

DHI: digital health intervention
HCD: human-centered design
PBD: person-based design
PCD: patient-centered design
PLD: patient-led design
PRISMA: Preferred Reporting Items for Systematic Reviews and Meta-Analyses
UCD: user-centered design
UX: user experience
